# Serum Ferritin Levels: A Potential Biomarker to Represent Child-Turcotte-Pugh Score among Decompensated Liver Cirrhosis Patients

**DOI:** 10.21315/mjms2019.26.2.7

**Published:** 2019-04-30

**Authors:** Taufik Sungkar, Muhammad Fakhrur Rozi, Leo Basa Dairi, Lukman Hakim Zain

**Affiliations:** 1 Department of Internal Medicine, Gastroenterology-Hepatology Division, Universitas Sumatera Utara, Padang Bulan, Medan, Indonesia; 2 Faculty of Medicine, Universitas Sumatera Utara, Padang Bulan, Medan, Indonesia

**Keywords:** inflammation, hepatitis, cirrhosis, prognosis, mortality

## Abstract

**Background:**

Liver cirrhosis and the child-Turcotte-Pugh (CTP) score are inseparable entities in liver disease. CTP score is largely known as the mortality and prognosis predictor. Nevertheless, ferritin emerges as a simple biomarker related to prognosis. The study aimed to determine whether there was a significant correlation between serum ferritin levels and CTP score.

**Methods:**

The study analysed 54 decompensated liver cirrhotic patients including 17 females and 37 males between May 2016 and May 2017 at the Haji Adam Malik General Hospital, Medan, Indonesia. Ferritin levels were, then, divided into trichotomous cut-off value (< 200 ng/mL, *n* = 22; 200–400 ng/mL, *n* = 5; and > 400 ng/mL, *n* = 27). Data was analysed using SPSS version 12.0 (continuous variables were assessed by the Kruskal-Wallis test and Chi-square test was used for categorical variables). In addition, Spearman correlation test was used to determine any significant correlation between ferritin levels and CTP score.

**Results:**

Based on data analysis, gender and CTP score were related to higher ferritin levels (*P* = 0.002 and *P* = 0.018, respectively). Furthermore, a significant correlation between serum ferritin levels and CTP score was obtained in to moderate degree (*P* = 0.000; *r* = 0.487).

**Conclusions:**

There might be a significant role of serum ferritin levels in predicting mortality and prognosis among decompensated liver cirrhosis patients but it still needs further attention.

## Introduction

Liver cirrhosis has been established as a condition where normal liver parenchyma was replaced with connective tissue producing nodule formation. Since it disrupts liver function, the term used to describe the condition is the end-stage of chronic liver disease. The etiologic factor can occur because of viral infection, excessive alcohol consumption or cryptogenic agent (no defining cause) (1, 2). In 2010, the disease contributed to 49,538 deaths of U.S. citizens with a male predominance (3). Meanwhile, there is no major difference compared with developing regions. Indonesia had 9% of citizens, or 1,284,000 people of the total viremic population with cirrhosis in 2014 and it is expected to increase to 15% in 2030. Although, Indonesia reduced its HBsAg endemicity to moderate level in 2013 but it still faces multiple problems related to cirrhotic complications. In addition, the number of decompensated liver cirrhotic patients is also expected to double to 19,400 patients by the end of year 2030 (4, 5).

The symptoms expand from compensated, no clinical manifestation, until decompensated stage where the complication including ascites, spontaneous bacterial peritonitis, hepatic encephalopathy or variceal bleeding could occur during the advance duration of the disease (1). Decompensated liver cirrhosis is strongly linked with high mortality rate. Therefore, using the scoring system to define mortality and prognosis is inevitable. Child-Turcotte-Pugh (CTP) score has been used for several years to predict prognosis and severity of liver cirrhosis patients. The scoring system consists of five indicators including albumin, international-normalised ratio (INR), bilirubin, ascites, and hepatic encephalopathy (6). Compared with model for end-stage liver disease (MELD) score, many studies have proved the capability of CTP score in predicting prognosis among diversed cirrhotic population, compensated and decompensated patients, and it does not need computational analysis. Therefore, bed-side interpretation can be carried out (7–9).

Ferritin is a 24-subunit protein, which is commonly used to represent iron levels in the human body indirectly but not in a liver cirrhotic patient (10). Nevertheless, the shifting use of ferritin levels for prognosis among of cirrhotic patients is emphasised in recent studies (11). High ferritin levels or hyperferritinemia might be related to poor prognosis of the chronic inflammatory disease as well as liver cirrhosis since its secretion can also depend on certain cytokines that have several roles during inflammatory surges. Furthermore, it is classified as an acute phase-reactant (12). In the initial findings, correlation between serum ferritin levels and the degree of inflammation was evident among patients with malignancy later its position helped to determine the clinical outcomes for liver cirrhotic patients (11). Since iron overload, in the first hint, indispensably induces an inflammatory cascade in the diseased liver, it relates to the disease progression and commonly occurs during viral hepatitis infection (13, 14). The study aimed to assess any significant correlation between ferritin level and CTP score. The study could provide further evidence that ferritin could be used to represent CTP score independently, to detect preventable circumstances. Demographic and laboratory findings were also differentiated based on the mean difference of serum ferritin levels.

## Material and Methods

### Patients

The cross-sectional study was carried out in one of tertiary referral hospital in the western part of Sumatera Island, Haji Adam Malik General Hospital, Medan, Indonesia, between May 2016 and May 2017. The non-random and consecutive sampling method was used to include suitable patients in the study based on their date of admission to the hospital. The exclusion criteria in the study were malignancy condition, or severe comorbidity, for instance end-stage renal disease and chronic pulmonary obstructive disorder, blood transfusion in the previous three months, positive HIV status, dyslipidemia and diabetes mellitus, acute liver failure, and pregnancy. Patients admitted to the internal medicine hospitalisation ward and diagnosed with decompensated liver cirrhosis were automatically considered for the study after satisfying the inclusion criteria and signing the written informed consent without coercion. Several laboratory findings were noted from the medical record registry, such as serum iron parameters, thrombocyte, international normalised ratio, bilirubin, serologic marker related to viral cirrhosis, and endoscopy for the presence of esophageal varices (15).

### CTP Score

The five indicators included in CTP score were assessed using physical and ultrasonography examination for ascites and encephalopathy while bilirubin, albumin, and INR were noted from the Haji Adam Malik medical record registry on the admission day. CTP score was calculated using the free online calculator provided by MdCacl (https://www.mdcalc.com/child-pugh-score-cirrhosis-mortality). CTP score was, then, divided into three class, A (5–6), B (7–9), and C (10–15). Thereafter, the mean difference of demographical characteristic comparison in each class and correlation analysis between serum ferritin and CTP score were carried out.

### Statistical analysis

The analysis was performed using Statistical Package for the Social Science (SPSS Inc., Chicago, IL) version 12.0 and depicted in percentage and medians or means with standard deviation. The data was not normally distributed, it was statistically proven based on the Kolmogorov-Smirnov normality test. Consequently, the data was analysed using non-parametric test (Kruskal-Wallis test). In exception to age variables, the data normal distribution was obtained; it was analysed using ANOVA test. The correlation between the serum ferritin level and CTP score was evaluated using Spearman correlation test (*P*-value < 0.05 was stated as significant results statistically with 95% confidence interval). Furthermore, the study was approved by the Medical Research Ethical Committee, Faculty of Medicine, Universitas Sumatera Utara, Medan, Indonesia (letter number: 544/TGL/KEPK FK USU-RSUPHAM/2017) and it was in accordance with the Declaration of Helsinki for medical research involving human subjects.

## Results

**Figure 1:**
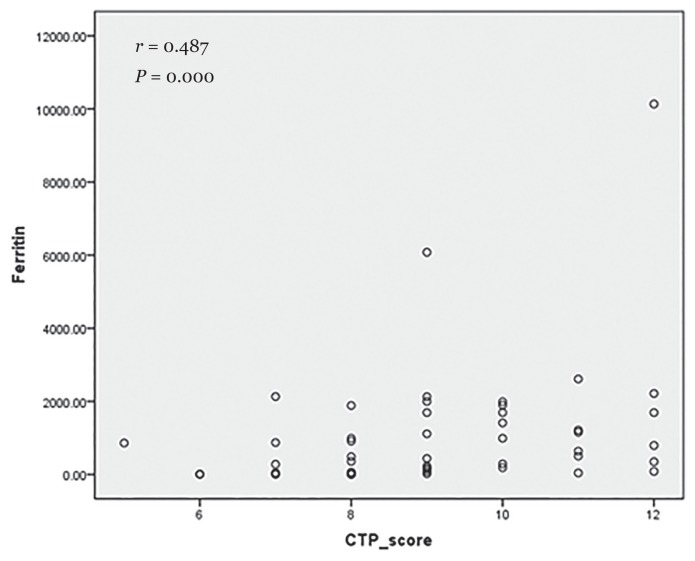
Moderate correlation between serum ferritin levels and CTP score among decompensated liver cirrhosis patients

The study enrolled 54 decompensated liver cirrhotic patients, 17 females and 37 males, with a mean age of 52.76 ± 12.57 years. Most patients had viral-related cirrhosis of hepatitis B and C since the serologic marker for viral infection was positive (HBsAg and anti-HCV) (*n* = 30 patients, non-hepatitis B and C patients were 24). The baseline characteristic was presented in Table 1 including several laboratory results and serum iron parameters and esophageal variceal findings. F3 Beppu classification for esophageal varices was the predominant grade (*n* = 22, 40.7%) followed by F2 (*n* = 16, 29.6%). In further analysis, the study obtained significant findings of mean difference using Kruskal-Wallis test, since the data was not distributed normally, such as gender and CTP score in accordance with serum ferritin (trichotomous cut-off values: ferritin under 200, 200–400, and over 400). In addition, creatinin and bilirubin levels were consistently and descriptively higher in patients with ferritin levels more than 400 μg/L. The other findings consisting of albumin, INR, and creatinin were also depicted in Table 1. Thus, the serum ferritin levels were significantly correlated with CTP score (*r* = 0.487; *P* = 0.000) (Figure 1).

## Discussion

In the study, the significant and positive correlation between serum ferritin levels and CTP score was evident among decompensated liver cirrhosis patients in the study. While, several laboratory findings tend to be varied with ferritin levels based on descriptive analysis, most findings had higher levels in accordance with high serum ferritin levels (> 400 μg/L), particularly CTP score. Therefore, it can be confirmed that a patient with hyperferritinemia has a tendency to have a higher CTP score with moderate correlation. Hepatocellular including chronic active hepatitis B and liver cirrhosis is the second most common etiology propagated by certain mechanism producing hyperferritinemia (16).

Iron overload adds the liver insults by inducing secretion of pro-inflammatory cytokine and producing inflammation and necrosis of liver cells (17). Ferritin as cytosolic protein egress into the vascular system as it leaks from necrotic liver cells, and hyperferritinemia will ensue. Therefore, the serum ferritin level, in this circumstance, is correlated with the extent of liver cell necrosis and it was proved by recent studies (18). In addition, liver biopsy was claimed as a golden standard to diagnose iron overload in liver tissue but it traditionally could produce complications outweighing its beneficial aspects, particularly among advanced-cirrhosis and low-platelet patients (19). In the circumstances, ferritin emerges as a biomarker used to predict severity of liver damage in chronic active liver disease (11). In addition, the samples included in the study mostly suffered from viral-related cirrhosis and there was evidence that viral infection induces disruption of iron hemostasis. However, the clear mechanism causing the condition has not been fully elucidated (20, 21). Gao et al. (22) give evidence, in addition to significant correlation between hyperferritinemia and CTP score, that hyperferritinemia was highly prevalent in hepatitis B-related liver disease patients.

Furthermore, several studies outlined the function of ferritin in predicting prognosis and severity of liver cirrhotic patients through longitudinal and retrospective study. Although this study used a small sample size, it proved the significant finding that ferritin was correlated with CTP score. Ripoll et al. (23) similarly found a significant correlation between hyperferritinemia and CTP score (*r* = 0.392, *P* = 0.009) by enrolling 51 patients with liver cirrhosis. In other perspectives, ferritin and CTP score were associated with poor prognosis through multivariate analysis among waiting list pre-transplant patients independently (24). Buyukasik et al. (25) showed that higher level of serum ferritin was confined to CTP class C patients (A/B/C= 198±25/161±161/366±396). Although, the relationship between ferritin and prognosis or the outcome still becomes inconsistent. There was a finding that ferritin could not be used solely to predict prognosis and the clinical outcome. Uchino et al. (26) stated that serum ferritin did not affect the prognosis among hepatocellular carcinoma patients who underwent radiofrequency ablation (RFA), in addition, there were lower serum ferritin levels among CTP score class C and it is more likely affected by tumor size and liver function (27).

The study also did not escape certain limitations. First, the causal relationship between ferritin and certain dependent variables could not be described since it was designed as a cross-sectional (point-time design). Second, serum ferritin levels would be affected by the presence of C282Y homozygosity producing iron overload (28) but the study did not perform the screening test to exclude the positive samples. Finally, the study also did not provide the output related to prognosis and outcome. Therefore, further studies exploring the accurate cut-off point of hyperferritinemia for certain poor clinical scenario should be carried out. One study had found that a significant cut-off value of serum ferritin levels (as much as 400 μg/L) could be used to predict one-month mortality in decompensated liver cirrhotic patients (29). In a larger study, 200 μg/L had been proved to predict 180-day and 1-year mortality among liver transplantation waiting list patients (30). Notwithstanding these findings, the precise cut-off value of serum ferritin remains uncertain.

## Conclusions

The study concluded that ferritin is an important biomarker that represents CTP score as an indirect scheme to predict prognosis and mortality among decompensated liver cirrhotic patients. Ferritin level is easily affected by several factors; therefore, longitudinal studies inevitably need to provide evidence of ferritin cut-off value related to prognosis and outcome since hyperferritinemia is a hallmark of liver inflammation instead of iron overload among decompensated cirrhotic patients.

## Figures and Tables

**Table 1:** Clinical characteristics and laboratory findings based on serum ferritin levels

Variables	Ferritin < 200 (*n* = 22)	Ferritin 200–400 (*n* = 5)	Ferritin > 400 (*n* = 27)	*P*-value
Age (years)^a^	52.55±13.15	58.4±11.19	51.89±12.50	0.574^d^
Gender				
Male/Female	15/7	0/5	22/5	0.002^*^
Albumin	2.45 (1.5–3.3)	1.9 (1.6–3.1)	2.2 (1.7–3.7)	0.419
INR^b^	1.28 (0.99–1.84)	1.54 (1.07–2.96)	1.32 (0.81–2.45)	0.266
Creatinine	0.9 (0.6–2.37)	0.88 (0.55–2.06)	1.18 (0.52–13.58)	0.198
Total bilirubin	1.01 (0.3–4.6)	1.9 (0.6–16.3)	2.41(0.29–29.8)	0.183
CTP score^c^	8 (6 12)	9 (7 12)	10 (5 12)	0.018^*^
CTP class (A/B/C)	1/18/3	0/3/2	1/12/14	0.089

The data is presented in

a mean (standard deviation) and median or interquartile range,

b International normalised ratio,

c Child-Turcotte-Pugh score,

d Age variable was analysed using ANOVA test, the rest variables using Kruskal-Wallis test

## References

[1] Wiegand J, Berg T (2013). The etiology, diagnosis and prevention of liver cirrhosis: part 1 of a series on liver cirrhosis. Dtsch Arztebl Int.

[2] Tsochatzis EA, Bosch J, Burroughs AK (2014). Liver cirrhosis. Lancet.

[3] Mokdad AA, Lopez AD, Shahraz S, Lozano R, Mokdad AH, Stanaway J (2014). Liver cirrhosis mortality in 187 countries between 1980 and 2010: a systematic analysis. BMC Med.

[4] Lusida MI, Juniastuti YY (2016). Current hepatitis B virus infection situation in Indonesia and its genetic diversity. World J Gastroenterol.

[5] Scrutton J, Wallace J, Wait S (2018). Situation analysis of viral hepatitis in Indonesia: a policy. [Internet].

[6] Garcia-Tsao G (2016). The child-Turcotte classification: from gestalt to sophisticated statistics and back. Dig Dis Sci.

[7] Hong SH, Kim JE, Cho ML, Heo YJ, Choi JH, Choi JH (2011). Comparison of the Child-Turcotte-Pugh classification and the model for end-stage liver disease score as predictors of the severity of the systemic inflammatory response in patients undergoing living-donor liver transplantation. J Korean Med Sci.

[8] Durand F, Valla D (2005). Assessment of the prognosis of cirrhosis: child–Pugh versus MELD. J Hepatol.

[9] Peng Y, Qi X, Guo X (2016). Child–Pugh versus MELD score for the assessment of prognosis in liver cirrhosis: a systematic review and metaanalysis of observational studies. Medicine.

[10] Knovich MA, Storey JA, Coffman LG, Torti SV, Torti FM (2009). Ferritin for the clinician. Blood reviews.

[11] Wang W, Knovich MA, Coffman LG, Torti FM, Torti SV (2010). Serum ferritin: past, present and future. Biochim Biophys Acta.

[12] Wessling-Resnick M (2010). Iron homeostasis and the inflammatory response. Ann Rev Nutr.

[13] Milic S, Mikolasevic I, Orlic L, Devcic E, Starcevic-Cizmarevic N, Stimac D (2016). The role of iron and iron overload in chronic liver disease. Medical Sci Monit.

[14] Koperdanova M, Cullis JO (2015). Interpreting raised serum ferritin levels. BMJ.

[15] Abby Philips C, Sahney A (2016). Oesophageal and gastric varices: historical aspects, classification and grading: everything in one place. Gastroenterol Report.

[16] Moore C, Ormseth M, Fuchs H (2013). Causes and significance of markedly elevated serum ferritin levels in an academic medical center. J Clin Rheumatol.

[17] Ramm GA, Ruddell RG (2005). Hepatotoxicity of iron overload: mechanisms of iron-induced hepatic fibrogenesis. Sem Liv Dis.

[18] Kowdley KV (2016). Iron overload in patients with chronic liver disease. Gastroenterol Hepatol [Internet].

[19] Seeff LB, Everson GT, Morgan TR, Curto TM, Lee WM, Ghany MG (2010). Complication rate of percutaneous liver biopsies among persons with advanced chronic liver disease in the HALT-C trial. Clin Gastroenterol Hepatol.

[20] Vagu C, Sultana C, Ruta S (2013). Serum iron markers in patients with chronic hepatitis C infection. Hepat Mon.

[21] Radicheva MP, Andonova AN, Milcheva HT, Ivanova NG, Kyuchukova SG, Nikolova MS (2018). Serum markers of iron metabolism in chronic liver diseases. Maced J Med Sci.

[22] Gao YH, Wang JY, Liu PY, Sun J, Wang XM, Wu RH (2018). Iron metabolism disorders in patients with hepatitis B-related liver diseases. World J Clin Cases.

[23] Ripoll C, Keitel F, Hollenbach M, Greinert R, Zipprich A (2015). Serum ferritin in patients with cirrhosis is associated with markers of liver insufficiency and circulatory dysfunction, but not of portal hypertension. J Clin Gastroenterol.

[24] Al-Freah MA, Kriese S, Foxton MR, Quaglia A, Bomford A, Heaton ND (2013). The association of pretransplant ferritin level with waiting list and post-transplant survival. Does ferritin actually predict outcome?. Transpl Int.

[25] Buyukasik NS, Nadir I, Akin FE, Cakal B, Kav T, Ersoy O (2011). Serum iron parameters in cirrhosis and chronic hepatitis: detailed description. Turk J Gastroenterol.

[26] Uchino K, Tateishi R, Nakagomi R, Fujiwara N, Minami T, Sato M (2018). Serum levels of ferritin do not affect the prognosis of patients with hepatocellular carcinoma undergoing radiofrequency ablation. PloS One.

[27] Wei Y, Ye W, Zhao W (2018). Serum iron levels decreased in patients with HBV-related hepatocellular carcinoma, as a risk factor for the prognosis of HBV-Related HCC. Front Physiol.

[28] Adams P (2008). Management of elevated serum ferritin levels. Gastroenterol Hepatol [Internet].

[29] Umer N, Makki MU, Kiran SK, Jadoon NA (2017). Serum ferritin as a predictor of 30 days mortality in patients of decompensated chronic liver disease. J Ayub Med Coll Abbottabad [Internet].

[30] Walker NM, Stuart KA, Ryan RJ, Desai S, Saab S, Nicol JA (2010). Serum ferritin concentration predicts mortality in patients awaiting liver transplantation. Hepatology.

